# A Web-Based Clinical System for Cohort Surveillance of Specific Clinical Effectiveness and Safety Outcomes: A Cohort Study of Non–Vitamin K Antagonist Oral Anticoagulants and Warfarin

**DOI:** 10.2196/13329

**Published:** 2019-07-03

**Authors:** Fong-Ci Lin, Shih-Tsung Huang, Rung Ji Shang, Chi-Chuan Wang, Fei-Yuan Hsiao, Fang-Ju Lin, Mei-Shu Lin, Kuan-Yu Hung, Jui Wang, Li-Jiuan Shen, Feipei Lai, Chih-Fen Huang

**Affiliations:** 1 Graduate Institute of Biomedical Electronics and Bioinformatics National Taiwan University Taipei Taiwan; 2 Department of Pharmacy National Taiwan University Hospital Taipei Taiwan; 3 Graduate Institute of Clinical Pharmacy, College of Medicine National Taiwan University Taipei Taiwan; 4 School of Pharmacy, College of Medicine National Taiwan University Taipei Taiwan; 5 Information Technology Office National Taiwan University Hospital Taipei Taiwan; 6 Department of Development and Planning National Taiwan University Hospital Taipei Taiwan; 7 Department of Internal Medicine National Taiwan University Hospital Taipei Taiwan; 8 Department of Internal Medicine National Taiwan University Hospital Hsinchu Taiwan; 9 Institute of Epidemiology and Preventive Medicine, College of Public Health National Taiwan University Taipei Taiwan; 10 Department of Computer Science & Information Engineering National Taiwan University Taipei Taiwan; 11 Department of Electrical Engineering National Taiwan University Taipei Taiwan

**Keywords:** public health surveillance, warfarin, anticoagulants, pharmacovigilance, drug safety

## Abstract

**Background:**

Conventional systems of drug surveillance lack a seamless workflow, which makes it crucial to have an active drug surveillance system that proactively assesses adverse drug events.

**Objective:**

The aim of this study was to develop a seamless, Web-based workflow for comparing the safety and effectiveness of drugs in a database of electronic medical records.

**Methods:**

We proposed a comprehensive integration process for cohort surveillance using the National Taiwan University Hospital Clinical Surveillance System (NCSS). We studied a practical application of the NCSS that evaluates the drug safety and effectiveness of novel oral anticoagulants (NOACs) and warfarin by cohort tree analysis in an efficient and interoperable platform.

**Results:**

We demonstrated a practical example of investigating the differences in effectiveness and safety between NOACs and warfarin in patients with nonvalvular atrial fibrillation (AF) using the NCSS. We efficiently identified 2357 patients with nonvalvular AF with newly prescribed oral anticoagulants between 2010 and 2015 and further developed 1 main cohort and 2 subcohorts for separately measuring ischemic stroke as the clinical effectiveness outcome and intracranial hemorrhage (ICH) as the safety outcome. In the subcohort of ischemic stroke, NOAC users exhibited a significantly lower risk of ischemic stroke than warfarin users after adjusting for age, sex, comorbidity, and comedication in an intention-to-treat (ITT) analysis (*P*=.01) but did not exhibit a significantly distinct risk in an as-treated (AT) analysis (*P*=.12) after the 2-year follow-up. In the subcohort of ICH, NOAC users did not exhibit a different risk of ICH both in ITT (*P*=.68) and AT analyses (*P*=.15).

**Conclusions:**

With a seamless and Web-based workflow, the NCSS can serve the critical role of forming associations between evidence and the real world at a medical center in Taiwan.

## Introduction

Although randomized controlled trials are considered the gold standard for the approval of new drugs, these trials may be ineffective in detecting adverse drug events (ADEs) in *real-world* clinical practice. Numerous drugs are withdrawn after market approval because of unexpected severe ADEs [1]. Many studies have indicated that the relatively small sample size of clinical trials compared with target patients in the real world is the major barrier to detecting very rare but serious or even fatal adverse events [2-4]. Therefore, it is critical to establish a well-designed, effective, and efficient active postmarketing drug surveillance system to continuously monitor and evaluate drug safety and effectiveness after a drug is launched.

In our previous study [5], we implemented a Web-based clinical surveillance system, the National Taiwan University Hospital (NTUH) Clinical Surveillance System (NCSS), which could integrate the workflow of cohort identification to accelerate the exploratory process of patients with specific disease diagnoses and medication usage patterns using electronic medical records (EMRs).

After cohort identification, the next obstacle to address, when using EMRs to implement a *real-world* postmarketing drug surveillance system, will be to reduce the differences between those who receive a specific drug and their comparators (the so-called *selection bias*). To avoid such bias, matching is one approach that can be followed to minimize the confounding effects resulting from such discrepancies [6,7]. In addition to matching, analytic tools such as regression modeling can also be used to remove these confounding effects and to adjust for imbalances between the treatment and the comparator groups [8].

In continuation with our previous efforts, we conducted this study with the aim of developing a Web-based outcome analysis module with a matching process to generate analysis-ready datasets. Canonical survival analysis methods and advanced statistical tests for comparing the safety and the effectiveness of drugs were also embedded in the system.

## Methods

### Workflow

**Figure 1:**
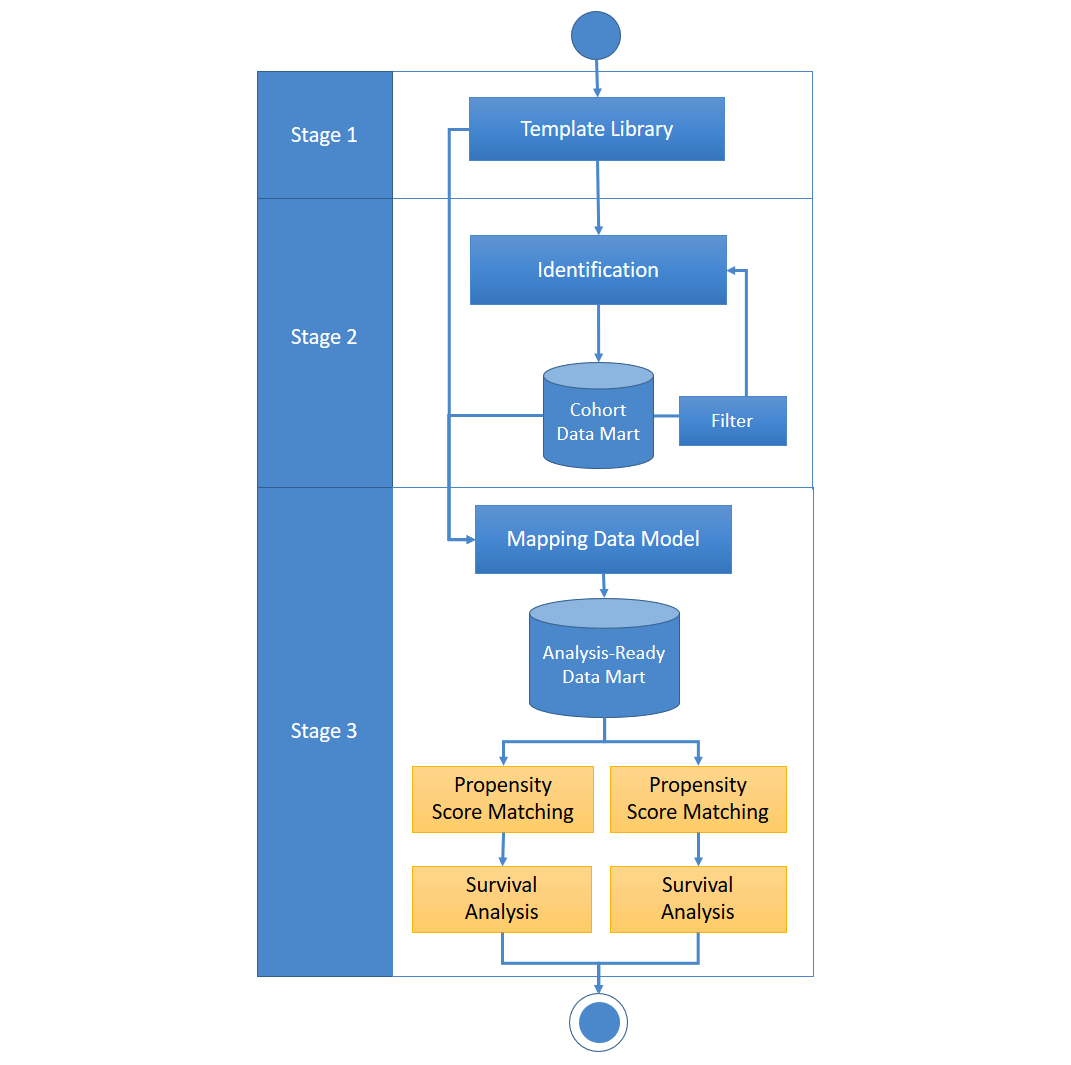
System workflow of the National Taiwan University Hospital Clinical Surveillance System.

Stages 1 and 2 were completed in our previous study [5]. We aimed to present the stage 3 cohort tree analysis for clinical surveillance in an efficient and interoperable platform that uses a secure https for all connections. The overall workflow of the NCSS is depicted in Figure 1.

### Stage 3: Cohort Tree Analysis

The Web interface is implemented in the ASP.Net framework (Microsoft, Redmond, WA) and R statistical environment designed for Web development and cloud batch processes. In this study, we focus on comparing the effectiveness of different treatments. We use new user cohort study design, which is conceptually similar to randomized controlled studies and widely used in observational studies. Moreover, the previous study also identified that the new user cohort study design is the primary design to be considered for studies of drug safety and comparative effectiveness [9]. Thus, we establish the structure of cohort tree analysis containing the following 3 processes as the stage 3 of our system: mapping data model, propensity score matching, and survival analysis.

#### Mapping Data Model

Survival analysis studies typically include a wealth of clinical, demographic, and biomarker information on patients and indicators for therapy or other interventions. If researchers seek to analyze multiple risk factors, they must perform preprocessing to map each variable to the study population.

We design an automated mechanism that can help the researcher generate analysis-ready datasets by combining covariables and demographic information from the database. First, researchers choose a study population from the cohort data mart and then define covariables or search existing templates from the template library. Second, the NCSS receives the request to automatically aggregate the analysis-ready dataset and deposit it to the analysis-ready data mart. Therefore, the analysis-ready data model can be reused again, reducing the computation overhead. Given this architecture, we can support complicated research situations, such as those that resemble a tree-like structure, so we named stage 3 *cohort tree analysis*.

#### Propensity Score Matching

A successful outcome analysis should ensure that confounding covariates are balanced between the distinct treatment groups [10]. The propensity score matching technique reduces the effects of confounding when using *real-world* data, such as EMRs, to estimate treatment effects [11]. In this process, the researchers could select an analysis-ready dataset from the analysis-ready data mart, and our system allowed the use of the logistic regression model to estimate the propensity score of each identified study subject. The NCSS uses the nearest neighbor matching [12] with the further restriction that the absolute difference in the propensity scores of matched subjects must be below the specified caliper distance. Finally, the NCSS provides the report of baseline characteristics of the study subjects, including before and after propensity score matching, for researchers to evaluate the impact of propensity score matching on minimizing selection bias.

#### Survival Analysis

In this process, we implemented 2 different types of outcome measurement methods: intention-to-treat (ITT) analysis and as-treated (AT) analysis [13-16]. The ITT analysis states that any subject should be analyzed as if the study subject had completely followed the original study design, which means the NCSS would not stop following up even when the subjects did not completely receive the treatment or control drug during the follow-up period. In contrast, the AT analysis states that the treatment assignment is based on the actual treatment the patients receive and not the treatment the patients are supposed to receive based on the original study design, which means the NCSS would stop following up when the patients stop treatment or control drug before the occurrence of the study outcome during the follow-up period.

Regarding statistical analysis methods, the NCSS provided 2 features, including the Kaplan-Meier survival plot and the multivariable Cox proportional hazards model, for survival analysis. The NCSS also embedded visualization functions via server-side R scripts using the *survival* package [17] and the *ggplot2* package [18]. The Kaplan-Meier survival plot is one of the statistical methods used to estimate the survival time after a period of treatment based on descriptive statistics. The multivariable Cox proportional hazards model is a statistical method for comparing the proportional effect of several risk factors on survival. In the model, the measurement of the effect is the hazard ratio (HR), which is the risk of failure, given that the participant has survived up to a specific time [19].

### Investigating the Clinical Effectiveness and Safety Between Non–Vitamin K Antagonist Oral Anticoagulants and Warfarin in Patients With Nonvalvular Atrial Fibrillation

In this section, we use an example to demonstrate the clinical application of the NCSS, which is used to investigate the clinical effectiveness and safety between novel oral anticoagulants (NOACs) and warfarin in patients with nonvalvular atrial fibrillation (AF). According to clinical guidelines [20,21], anticoagulant therapy is recommended for AF patients to prevent the risk of ischemic stroke, which is one of the major complications of AF. Warfarin, a non–vitamin K antagonist, was the only option for oral anticoagulant treatment in AF patients for decades. Although warfarin is an effective treatment for ischemic stroke prevention, its therapeutic effect is complicated because of a narrow therapeutic range and multiple drug-food and drug-drug interactions [22-24]. These features led to a requirement for monitoring to optimize the therapeutic dose to prevent the risk of adverse events, especially major bleeding [22,23].

In recent years, the NOACs (ie, dabigatran, rivaroxaban, and apixaban) have been launched and suggested as alternatives to warfarin. Compared with warfarin, NOACs demonstrated similar or better stroke prevention effects and similar or lower risks of bleeding in clinical trials [25-27]. Moreover, the NOACs exhibit fewer drug-food or drug-drug interactions and do not require regular monitoring. Although the effectiveness and safety of NOACs have been proven in clinical trials, whether these effects observed in clinical trials translate well in *real-world* clinical practice has not been discussed. We aimed to investigate the clinical effectiveness and safety between NOACs and warfarin in patients with nonvalvular AF within the NTUH clinical surveillance system. The details of clinical orders for inclusion criteria, exclusion criteria, outcome measures, comedication, and comorbidities are presented in Multimedia Appendix 1 **.**

### Study Population

We first identified patients with AF who were aged at least 20 years, but without a diagnosis of prosthetic heart valve or mitral valve disease between 2010 and 2015, as our study cohort. We further identified subjects who were newly prescribed anticoagulants, including warfarin or NOACs, during the study period. The first date of anticoagulant prescription was defined as the index date for each study subject. The subjects who had ever received any anticoagulant prescription or who were pregnant, diagnosed with cancer, or under chronic dialysis within 1 year before the index date were excluded. We also excluded subjects prescribed NOACs along with warfarin on the index date.

The outcomes of interest, including ischemic stroke and intracranial hemorrhage (ICH), were irreversible events. To ensure that these irreversible outcomes that occurred during the follow-up period were incident events, which refer to new events, we identified 2 subcohorts, excluding those who had the irreversible outcomes within 1 year before the index date, and conducted statistical analysis separately. Finally, we stratified the subjects into 2 study groups, NOACs and warfarin users, in each subcohort.

### Outcome Measures

The outcomes of interest in this study were clinical effectiveness and safety. Clinical effectiveness was defined as ischemic stroke. Safety was defined as ICH. These outcomes were assessed separately in the above-mentioned subcohorts during the follow-up period. Any diagnoses in the records of outpatient visits, hospitalization, and emergency room visits were applied for the assessment of the study outcomes.

In this practical example of the NCSS, we used both ITT and AT analyses. In ITT analysis, patients were followed from the index date to the following events: (1) occurrence of the outcome of interest or (2) the end of a 2-year follow-up since the index date, whichever came first. In the AT analysis, patients were followed from the index date to the following events: (1) occurrence of the outcome of interest, (2) discontinuation of the index anticoagulant, or (3) the end of a 2-year follow-up since the index date, whichever came first. Medication discontinuation was defined as either discontinuing oral anticoagulation therapy or having a greater than 30-day gap between the end of an oral anticoagulant prescription and the next prescription.

### Covariates

The covariates adjusted were those known to affect anticoagulant treatment and study outcomes, including age, gender, annual stroke risk, specific comorbidities, and concomitant medications. Comorbidities were identified by diagnoses made within 12 months before the index date. Concomitant medications were identified by at least one prescription within 12 months preceding the index date.

### Statistical Analysis

One-to-one propensity score matching using a nearest neighbor matching algorithm with a maximum matching caliper of 0.2 was applied to balance the covariates of baseline characteristics between the NOAC and warfarin groups. The absolute standardized mean differences were applied to compare the between-group differences of the baseline characteristics. An absolute standardized difference of less than 0.1 was recognized as indicating no significant difference. Two kinds of survival analysis, Kaplan-Meier curve and Cox proportional hazard model, were applied to assess the relationship between anticoagulant treatment and study outcomes. In addition, 2-sided tests with *P*<.05 were defined as statistically significant.

## Results

**Figure 2:**
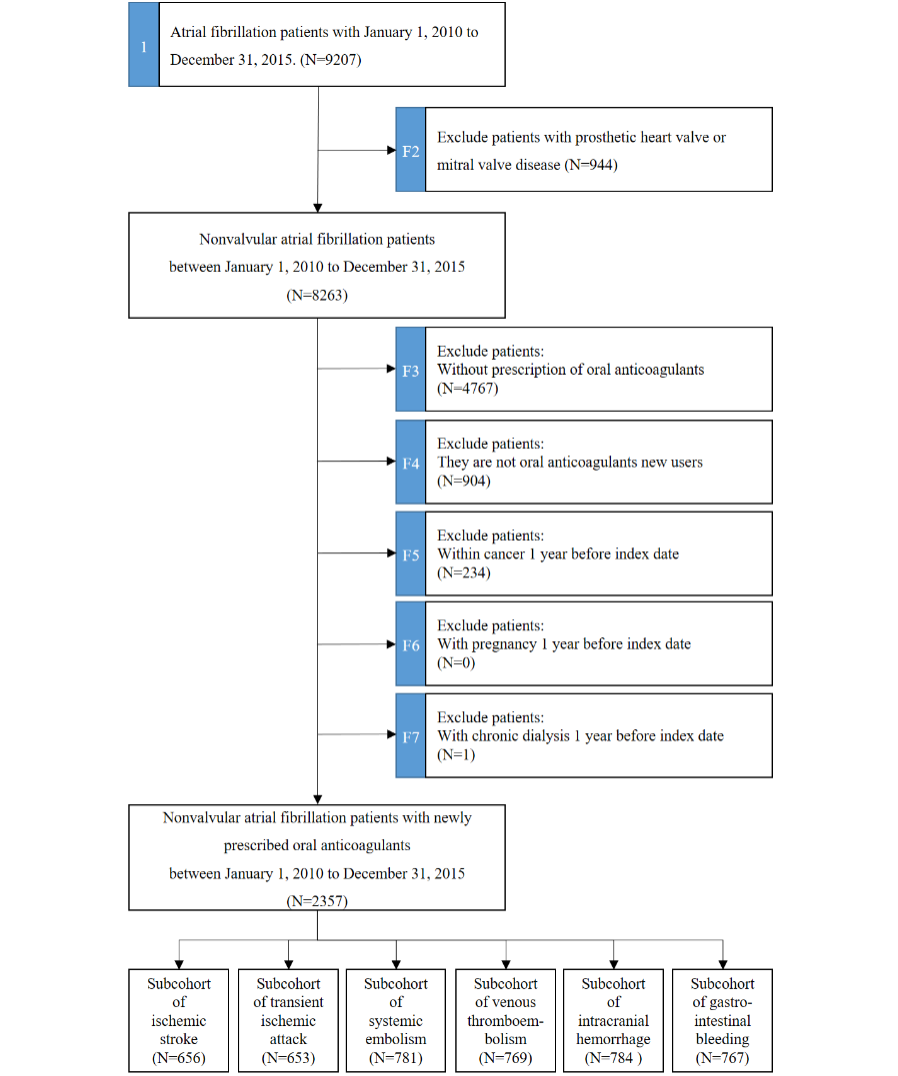
Study flowchart implemented by the National Taiwan University Hospital Clinical Surveillance System. This study flow contains 7 identification processes. Each identification process was assigned a universally unique identifier with a case number (marked by the blue background, such as 1, F2, F3, F4, F5, F6, and F7).

We demonstrated a practical example of investigating the clinical effectiveness and safety between NOACs and warfarin in patients with nonvalvular AF and implemented the hierarchical study population using the NCSS, as depicted in Figure 1. We initially identified 9207 AF patients who were aged 20 years or older between 2010 and 2015. Approximately 89.74% (8263/9207) of these patients were nonvalvular AF patients. By adopting the identification and filter function of the NCSS, patients without an oral anticoagulant prescription during the study period (n=4767), those with cancer (n=234), those who were pregnant (n=0), or those undergoing chronic dialysis (n=1) within 1 year before the index date were excluded. In addition, to identify new oral anticoagulants users, we excluded 907 patients with an oral anticoagulants prescription before the index date. Overall, we identified 2357 patients with AF, who were newly prescribed oral anticoagulants between 2010 and 2015, as our study subjects. The study flowchart of the NCSS is depicted in Figure 2.

**Figure 3:**
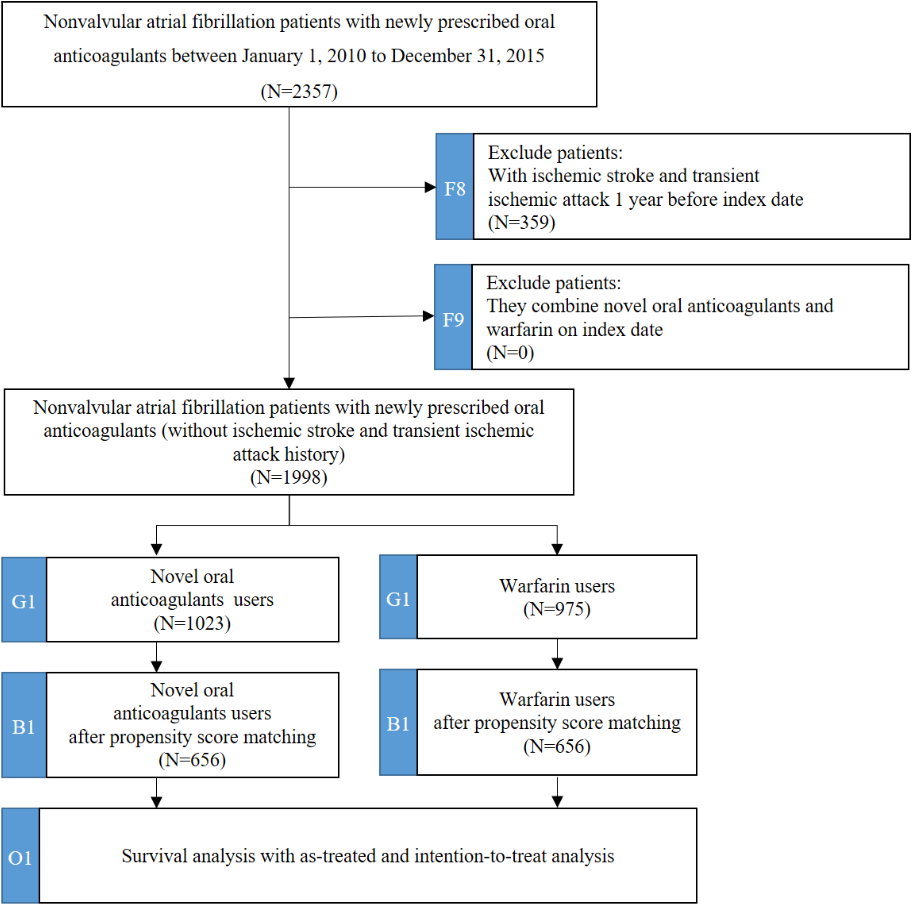
The study flow of subcohort of ischemic stroke. The subcohort contains 2 identification processes (F8 and F9), 1 mapping data model process (G1), 1 propensity score matching process (B1), and 1 survival analysis process (O1).

After cohort identification, we further examined the 2 subcohorts to analyze ischemic stroke and ICH. In the subcohort of ischemic stroke, we further excluded subjects who experienced ischemic stroke or transient ischemic attack (TIA) within 1 year before the index date (n=359) from the original cohort and categorized them into the NOAC group (n=1023) and the warfarin group (n=975) according to their first use of oral anticoagulants at the index date. After propensity score matching, the final sample included 656 NOAC-warfarin matched pairs. The study flow of subcohort of ischemic stroke is depicted in Figure 3.

**Figure 4:**
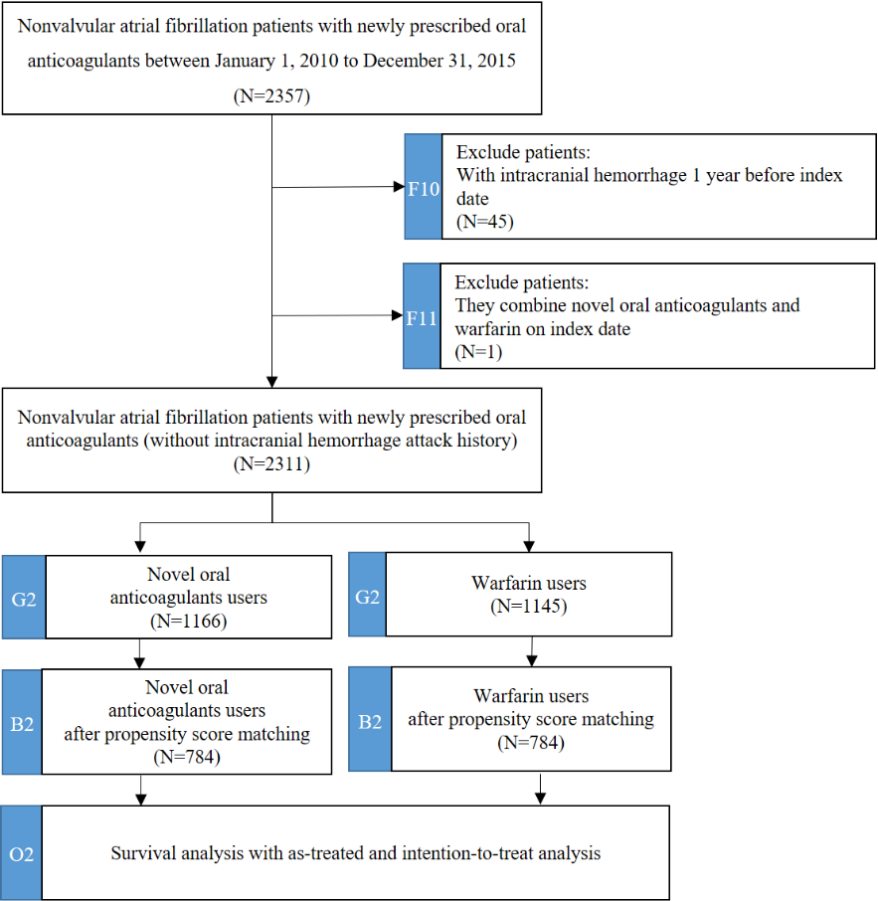
The study flow of subcohort for intracranial hemorrhage. The subcohort contains 2 identification processes (F10 and F11), 1 mapping data model process (G2), 1 propensity score matching process (B2), and 1 survival analysis process (O2).

In the subcohort for ICH, we further excluded subjects who experienced ischemic stroke or TIA within 1 year before the index date (n=45) and subjects prescribed NOACs along with warfarin on the index date (n=1) from the original cohort. We categorized these subjects into the NOAC group (n=1166) and the warfarin group (n=1145) based on the first oral anticoagulant used at the index date. After propensity score matching, the final sample contained 784 NOAC-warfarin matched pairs. The study flow of subcohort of ICH is depicted in Figure 4.

All of the standardized mean differences in each variable were less than 0.1, revealing a good between-group balance of baseline characteristics. The details of baseline characteristics before and after matching are presented in Multimedia Appendix 1.

Table 1 shows that warfarin users exhibited the higher crude incidence rates of ischemic stroke both in the ITT analysis (warfarin: 6.26 events per 100,000 patient-years; NOACs: 2.82 events per 100,000 patient-years) and AT analysis (warfarin: 10.68 events per 100,000 patient-years; NOACs: 6.57 events per 100,000 patient-years) after 2 years of follow-up. However, warfarin users exhibited lower crude incidence rates of ICH both in ITT analysis (warfarin: 0.43 events per 100,000 patient-years; NOACs: 1.91 events per 100,000 patient-years) and AT analysis (warfarin: 0.54 events per 100,000 patient-years; NOACs: 0.72 events per 100,000 patient-years) after 2 years of follow-up. Kaplan-Meier survival plot results are displayed in Multimedia Appendix 1.

The results of the adjusted Cox proportional hazards models are summarized in Table 2. After the 2-year follow-up, NOAC users exhibited a significantly lower risk of ischemic stroke than warfarin users after adjusting for age, sex, comorbidity, and comedication in ITT analysis (adjusted HR=0.41, *P*=.01) but did not exhibit a significant difference of risk in AT analysis (adjusted HR=0.54, *P*=.12). Regarding ICH, NOAC users did not exhibit a significantly distinct risk of ICH both in ITT analysis (adjusted HR=1.42, *P*=.68) and AT analysis (adjusted HR=254.15, *P*=.15).

**Table 1:** The incidence of ischemic stroke and intracranial hemorrhage (N=2357).

Method	Group	Outcome	Events	Follow-up duration (patient-days)	Incidence density	Cumulative incidence, % (95% CI)
ITT^a^ analysis	Warfarin	Ischemic stroke (n=656)	28	446,943	6.26	4.27 (2.84-6.17)
ITT analysis	NOAC^b^	Ischemic stroke (n=656)	13	461,354	2.82	1.98 (1.06-3.39)
AT^c^ analysis	Warfarin	Ischemic stroke (n=656)	19	177,980	10.68	2.90 (1.74-4.52)
AT analysis	NOAC	Ischemic stroke (n=656)	11	167,508	6.57	1.68 (0.84-3.00)
ITT analysis	Warfarin	Intracranial hemorrhage (n=784)	3	550,999	0.54	0.38 (0.08-1.12)
ITT analysis	NOAC	Intracranial hemorrhage (n=784)	4	556,467	0.72	0.51 (0.14-1.31)
AT analysis	Warfarin	Intracranial hemorrhage (n=784)	1	230,972	0.43	0.13 (0.00-0.71)
AT analysis	NOAC	Intracranial hemorrhage (n=784)	4	208,929	1.91	0.51 (0.14-1.31)

^a^ITT: intention-to-treat.

^b^NOAC: novel oral anticoagulants.

^c^AT: as-treated.

**Table 2:** The hazard ratio of ischemic stroke and intracranial hemorrhage (N=2357).

Method	Group	Outcome	Events	Hazard ratio	*P* value	95% CI
ITT^a^ analysis	Warfarin	Ischemic stroke (n=656)	28	1	—^b^	—
ITT analysis	NOAC^c^	Ischemic stroke (n=656)	13	0.41	0.01	0.21-0.82
AT^d^ analysis	Warfarin	Ischemic stroke (n=656)	19	1	—	—
AT analysis	NOAC	Intracranial hemorrhage (n=656)	11	0.54	0.12	0.25-1.16
ITT analysis	Warfarin	Ischemic stroke (n=784)	3	1	—	—
ITT analysis	NOAC	Intracranial hemorrhage (n=784)	4	1.42	0.68	0.26-7.82
AT analysis	Warfarin	Ischemic stroke (n=784)	1	1	—	—
AT analysis	NOAC	Intracranial hemorrhage (n=784)	4	254.16	0.15	0.16-478097.30

^a^ITT: intention-to-treat.

^b^Not applicable.

^c^NOAC: novel oral anticoagulants.

^d^AT: as-treated.

## Discussion

### Principal Findings

The results of this study confirm that the NCSS is a feasible and useful approach to enable systematic analysis for evaluating the clinical effectiveness and safety of drugs for clinical needs. We have successfully demonstrated the implementation of an application for assessing the clinical effectiveness and safety of NOACs and warfarin. To the best of our knowledge, the NCSS is a pioneering Web-based clinical surveillance system in Taiwan.

Through this practical example, we found that NOAC users exhibited a significantly lower risk of ischemic stroke than warfarin users but did not have a different risk of ICH in the ITT analysis. This result regarding clinical effectiveness was very similar to that reported in the pivotal clinical trials of NOACs and some of the observational studies with an ITT design for an outcome approach [25,26]. Regarding AT analysis, we found that both the risk of ischemic stroke and ICH were similar between NOAC and warfarin users. Given that the AT analysis states that the treatment assignment is based on the actual treatment the patients receive, patients who discontinued their index anticoagulants stopped follow-up, and their data were censored [13-16]. The definition of treatment exposure is more close to the real-world situation, in which patients may discontinue or change their treatment. However, the total follow-up time and frequency of the events in the AT analysis are less compared with ITT analysis. AT analysis may not have sufficient statistical power to test the hypothesis, especially when the outcome is a rare event. In our practical example, only 1 ICH event occurred in the warfarin group, so the HR is extremely large (adjusted HR=254.15, *P*=.15) but lacks statistical significance given the insufficient statistical power.

There are several important core concepts in our research, including designing the seamless workflow for active drug surveillance that enables a quick response in each step of the automatic process for statistical analysis. Although previous studies [28-31] have sought to generate similar systems, they have not proposed how to integrate a Web-based interoperable and user-friendly platform in designing a drug surveillance analysis. Most of the existing systems are based on offline operations using SAS software (SAS Institute), such as Sentinel [29] and AsPEN [28], in which researchers have developed a series of macros for distributed databases. Thus, the researcher who seeks to use the tool must first preprocess the data by himself or herself, but analyzing the large volume of data requires numerous resources and technical skills [32]. These technical gaps thus hinder the feasibility of conducting timely clinical research and delay the application of research results that would improve clinical practice. The NCSS has a highly integrated platform, in which workflows of clinical surveillance analysis can accelerate the survey process.

Another strength of our NCSS system is that we have built a highly reusable infrastructure for evaluation of the clinical effectiveness and safety in multiple subcohorts that most existing studies [33] have not considered. With our newly developed architecture of the stage 3 cohort tree analysis in the NCSS, the NCSS currently offers powerful features for statistical inferences, statistical adjustment for confounding factors, data preprocessing, and data visualization and generates risk effect estimates. This integrated solution allows the dynamic generation of multiple analysis-ready datasets in data mart and reduces the computational overhead through the reuse of the similar research design. This mechanism can inspire researchers and support more efficient outcome validation rather than data processing.

In summary, by generating an integrated survival analysis workflow to achieve the following targets, this study solves the following bottlenecks in constructing a timely postmarketing surveillance system. First, regarding accessibility, we designed the tool to be as straightforward as possible to reduce the learning threshold of clinical studies. Second, regarding efficiency, the NCSS is a Web-based application that can quickly respond in each step automatically to process statistical analysis. Third, regarding outcomes assessed in inferential analyses, the NCSS allows researchers to identify medical conditions defined as outcomes of interest in inferential analyses and their respective code lists and algorithm criteria. The NCSS will not only help researchers in the field of outcome research to analyze their data in depth but will also potentially facilitate the standardization of survival analysis at a medical center in Taiwan.

### Limitations

This study has some limitations that should be addressed. First, we build up the NCSS system at only 1 medical center in Taiwan. For acute diseases, patients may be treated in a nearby hospital. If these acute diseases happen to be rare events, the NCSS would not be able to detect the risk signal. However, the results of this study may likely be generalized to other medical centers with features similar to our medical center. Second, the use of diagnosis codes to identify the study cohort relied on the quality of coding in the hospital. A previous study [34] demonstrates that the medical center typically had better coding quality than the district hospital, and all the hospitals must pass the same level of accreditation with the National Health Insurance Administration in Taiwan. Third, the current NCSS only automatically extracts structured data in EMRs. Deep learning offers many opportunities for natural language processing and image classification [35,36]. In fact, some quality measures that use only unstructured data from the EMRs are relatively difficult to automate. Some unstructured data, such as ultrasound reports or x-ray reports, still currently use free text. Therefore, most clinical studies mainly use structured data for research. Future studies may consider combining unstructured data for clinical research.

### Conclusions

As of now, the NCSS is well constructed and continuously improving. Our teams consist of individuals in multidisciplinary specialties, such as clinical doctors, pharmacists, biomedical engineers, and epidemiologists. Several research teams have used the NCSS to enhance the research process based on their relevant clinical needs. An evaluation of the longitudinal trends of health care utilization can help create the baseline, track progress over time, and generate real-world evidence. The NCSS can serve the critical role of forming associations between evidence derived from clinical trials and the real world in a rapid fashion.

## Appendix

Multimedia Appendix 1 Supplement of investigating the clinical effectiveness and safety between novel oral anticoagulants and warfarin.

## Abbreviations

ADE: adverse drug event
AF: atrial fibrillation
AT: as-treated
EMRs: electronic medical records
HR: hazard ratio
ICH: intracranial hemorrhage
ITT: intention-to-treat
NCSS: National Taiwan University Hospital Clinical Surveillance System
NOACs: novel oral anticoagulants
NTUH: National Taiwan University Hospital
TIA: transient ischemic attack
