# Supplementary material for: A Web-Based Clinical System for Cohort Surveillance of Specific Clinical Effectiveness and Safety Outcomes: A Cohort Study of Non–Vitamin K Antagonist Oral Anticoagulants and Warfarin
Source: JMIR Med Inform. 2019 Jul 3;7(3):e13329. doi: 10.2196/13329 (PMC6636345; doi:10.2196/13329)
Supplement: Multimedia Appendix 1 [file medinform_v7i3e13329_app1.pdf]

Appendix: Supplement of NOAC and warfarin study

Table A1. ATC codes of the medications 2

Table A2. Exclusion criteria 3

Table A3. ICD-9-CM codes for outcome measures 4

Figure A1.Basic characteristics of the sub-cohort of ischemic stroke before Propensity Score Matching........ 5

Figure A2. Basic characteristics of the sub-cohort of intracranial hemorrhage after Propensity Score Matching. 6

Figure A3. Basic characteristics of the sub-cohort of intracranial hemorrhage after Propensity Score Matching. 7

Figure A4. Basic characteristics of the sub-cohort of ischemic stroke after Propensity Score Matching. 8

Figure A5. Kaplan–Meier Survival Plots of ischemic stroke with ITT analysis 9

Figure A6. Kaplan–Meier Survival Plots of ischemic stroke with AT analysis 10

Figure A7. Kaplan–Meier Survival Plots of Intracranial hemorrhage with ITT analysis 11

Figure A8. Kaplan–Meier Survival Plots of Intracranial hemorrhage with AT analysis 12

Table A1. ATC codes of the medications

**Medications**	**ATC code**
Warfarin	B01AA03
Rivaroxaban	B01AF01
Dabigatran	B01AE07
Apxiaban	B01AF02
Antiplatelet drugs	B01AC
Proton-pump inhibitor	A02BC
H2 receptor antagonist	A02BA
Other antacids	A02A
NSAIDs	M01A
Antiarrhythmic drugs	C01B
Digoxin	C01AA05
Beta-blockering agents	C07A
Dihydropyridine calcium channel blockers	C08C
Non-dihydropyridine calcium channel	C08D
Statins	C10AA
Anti-diabetes drugs	A10
ARBs/ACEIs	C09

Table A2. Exclusion criteria

Diagnosis	ICD-9-CM codes and diagnosis
Prosthetic heart valve	V42.2 heart valve replaced by transplant<br>V43.3 heart valve replaced by a mechanical device<br>353.0 closed heart valvotomy<br>35.2 open and other replacement of heart valve
Heart valve related disorder	394 disease of mitral valve<br>396 disease of mitral and aortic valves<br>424.0 mitral valve disorder
Pregnancy	V22 Normal pregnancy
Chronic dialysis	V45.1 postsurgical renal dialysis status<br>V56 encounter for dialysis and dialysis catheter care<br>39.95 hemodialysis<br>54.98 peritoneal dialysis
Cancer	140–149 Malignant neoplasm of lip, oral cavity, and pharynx<br>150–159 Malignant neoplasm of digestive organs and peritoneum<br>160–165 Malignant neoplasm of respiratory and intrathoracic organs<br>170–175 Malignant neoplasm of bone, connective tissue, skin, and breast<br>176–176 Kaposi's sarcoma<br>179–189 Malignant neoplasm of genitourinary organs<br>190–199 Malignant neoplasm of other and unspecified sites<br>200–208 Malignant neoplasm of lymphatic and hematopoietic tissue

Table A3. ICD-9-CM codes for outcome measures

**Outcomes**	**ICD-9-CM codes and diagnosis**
Ischemic stroke	433 Occlusion and stenosis of prevertebral arteries with cerebral infarction<br>434 Occlusion of cerebral arteries with cerebral infarction
Intracranial Hemorrhage	430 Subarachnoid hemorrhage<br>431 Intra-cerebral hemorrhage<br>432 Other and unspecified intracranial hemorrhage

Before Propensity Score Matching

	NOAC	Warfarin	SMD
n	1023	975	
ARBs/ACEIs = 1 (%)	481 (47.0)	378 (38.8)	0.167
Acute myocardial infarction = 1 (%)	24 (2.3)	12 (1.2)	0.084
Age 65-69 = 1 (%)	166 (16.2)	138 (14.2)	0.058
Age 70-74 = 1 (%)	197 (19.3)	112 (11.5)	0.217
Age 75-79 = 1 (%)	208 (20.3)	123 (12.6)	0.209
Age <65 = 1 (%)	133 (13.0)	418 (42.9)	0.706
Age >=80 = 1 (%)	319 (31.2)	184 (18.9)	0.287
Antiplatelet drugs = 1 (%)	524 (51.2)	374 (38.4)	0.261
Coagulation deficiency = 1 (%)	1 (0.1)	2 (0.2)	0.028
Diabetes = 1 (%)	236 (23.1)	168 (17.2)	0.146
Digoxin = 1 (%)	124 (12.1)	131 (13.4)	0.039
Dihydropyridine calcium channel blockers = 1 (%)	356 (34.8)	257 (26.4)	0.184
GI bleeding = 1 (%)	25 (2.4)	23 (2.4)	0.006
Gender = 1 (%)	427 (41.7)	378 (38.8)	0.061
H2 receptor antagonist = 1 (%)	105 (10.3)	87 (8.9)	0.046
Heart failure = 1 (%)	176 (17.2)	162 (16.6)	0.016
Hypertension = 1 (%)	514 (50.2)	406 (41.6)	0.173
Intracranial hemorrhage = 1 (%)	7 (0.7)	4 (0.4)	0.037
Liver disease = 1 (%)	34 (3.3)	45 (4.6)	0.066
NSAIDs = 1 (%)	150 (14.7)	136 (13.9)	0.020
Non-dihydropyridine calcium channel blockers = 1 (%)	147 (14.4)	133 (13.6)	0.021
Other antacids = 1 (%)	275 (26.9)	273 (28.0)	0.025
Peptic ulcer disease = 1 (%)	54 (5.3)	43 (4.4)	0.040
Peripheral vascular disease = 1 (%)	16 (1.6)	17 (1.7)	0.014
Proton-pump inhibitor = 1 (%)	98 (9.6)	120 (12.3)	0.087
Renal disease = 1 (%)	25 (2.4)	69 (7.1)	0.219
Statins = 1 (%)	240 (23.5)	160 (16.4)	0.177
Venous Thromboembolism = 1 (%)	9 (0.9)	12 (1.2)	0.034
anti-diabetes drugs = 1 (%)	191 (18.7)	155 (15.9)	0.073
antiarrhythmic drugs = 1 (%)	388 (37.9)	393 (40.3)	0.049
beta-blockering agents = 1 (%)	449 (43.9)	431 (44.2)	0.006

Figure A1.Basic characteristics of the subcohort of ischemic stroke before Propensity Score Matching.

After Propensity Score Matching

	NOAC	Warfarin	SMD
n	656	656	
ARBs/ACEIs = 1 (%)	266 (40.5)	265 (40.4)	0.003
Acute myocardial infarction = 1 (%)	10 (1.5)	10 (1.5)	<0.001
Age 65-69 = 1 (%)	122 (18.6)	126 (19.2)	0.016
Age 70-74 = 1 (%)	113 (17.2)	106 (16.2)	0.029
Age 75-79 = 1 (%)	118 (18.0)	114 (17.4)	0.016
Age <65 = 1 (%)	132 (20.1)	139 (21.2)	0.026
Age >=80 = 1 (%)	171 (26.1)	171 (26.1)	<0.001
Antiplatelet drugs = 1 (%)	256 (39.0)	267 (40.7)	0.034
Coagulation deficiency = 1 (%)	1 (0.2)	2 (0.3)	0.032
Diabetes = 1 (%)	131 (20.0)	127 (19.4)	0.015
Digoxin = 1 (%)	84 (12.8)	95 (14.5)	0.049
Dihydropyridine calcium channel blockers = 1 (%)	191 (29.1)	192 (29.3)	0.003
GI bleeding = 1 (%)	19 (2.9)	14 (2.1)	0.049
Gender = 1 (%)	274 (41.8)	266 (40.5)	0.025
H2 receptor antagonist = 1 (%)	60 (9.1)	62 (9.5)	0.010
Heart failure = 1 (%)	104 (15.9)	117 (17.8)	0.053
Hypertension = 1 (%)	295 (45.0)	298 (45.4)	0.009
Intracranial hemorrhage = 1 (%)	5 (0.8)	4 (0.6)	0.018
Liver disease = 1 (%)	26 (4.0)	22 (3.4)	0.032
NSAIDs = 1 (%)	100 (15.2)	102 (15.5)	0.008
Non-dihydropyridine calcium channel blockers = 1 (%)	85 (13.0)	89 (13.6)	0.018
Other antacids = 1 (%)	202 (30.8)	194 (29.6)	0.027
Peptic ulcer disease = 1 (%)	34 (5.2)	35 (5.3)	0.007
Peripheral vascular disease = 1 (%)	11 (1.7)	15 (2.3)	0.044
Proton-pump inhibitor = 1 (%)	81 (12.3)	80 (12.2)	0.005
Renal disease = 1 (%)	24 (3.7)	23 (3.5)	0.008
Statins = 1 (%)	106 (16.2)	121 (18.4)	0.060
Venous Thromboembolism = 1 (%)	6 (0.9)	8 (1.2)	0.030
anti-diabetes drugs = 1 (%)	112 (17.1)	104 (15.9)	0.033
antiarrhythmic drugs = 1 (%)	245 (37.3)	246 (37.5)	0.003
beta-blockering agents = 1 (%)	276 (42.1)	279 (42.5)	0.009

Figure A2. Basic characteristics of the subcohort of ischemic stroke after Propensity Score Matching.

Before Propensity Score Matching

	NOAC	Warfarin	SMD
n	1166	1145	
ARBs/ACEIs = 1 (%)	548 (47.0)	455 (39.7)	0.147
Acute myocardial infarction = 1 (%)	25 (2.1)	14 (1.2)	0.072
Age 65-69 = 1 (%)	188 (16.1)	160 (14.0)	0.060
Age 70-74 = 1 (%)	222 (19.0)	144 (12.6)	0.178
Age 75-79 = 1 (%)	231 (19.8)	148 (12.9)	0.187
Age <65 = 1 (%)	159 (13.6)	470 (41.0)	0.646
Age >=80 = 1 (%)	366 (31.4)	223 (19.5)	0.276
Antiplatelet drugs = 1 (%)	630 (54.0)	494 (43.1)	0.219
Coagulation deficiency = 1 (%)	1 (0.1)	3 (0.3)	0.042
Diabetes = 1 (%)	265 (22.7)	206 (18.0)	0.118
Digoxin = 1 (%)	136 (11.7)	168 (14.7)	0.089
Dihydropyridine calcium channel blockers = 1 (%)	419 (35.9)	335 (29.3)	0.143
GI bleeding = 1 (%)	29 (2.5)	29 (2.5)	0.003
Gender = 1 (%)	497 (42.6)	444 (38.8)	0.078
H2 receptor antagonist = 1 (%)	133 (11.4)	115 (10.0)	0.044
Heart failure = 1 (%)	188 (16.1)	183 (16.0)	0.004
Hypertension = 1 (%)	575 (49.3)	477 (41.7)	0.154
Ischemic stroke = 1 (%)	94 (8.1)	90 (7.9)	0.007
Liver disease = 1 (%)	38 (3.3)	50 (4.4)	0.058
NSAIDs = 1 (%)	191 (16.4)	176 (15.4)	0.028
Non-dihydropyridine calcium channel blockers = 1 (%)	187 (16.0)	193 (16.9)	0.022
Other antacids = 1 (%)	357 (30.6)	394 (34.4)	0.081
Peptic ulcer disease = 1 (%)	61 (5.2)	48 (4.2)	0.049
Peripheral vascular disease = 1 (%)	33 (2.8)	35 (3.1)	0.013
Proton-pump inhibitor = 1 (%)	169 (14.5)	210 (18.3)	0.104
Renal disease = 1 (%)	28 (2.4)	76 (6.6)	0.205
Statins = 1 (%)	296 (25.4)	222 (19.4)	0.144
Transient ischemic attack = 1 (%)	17 (1.5)	22 (1.9)	0.036
Venous Thromboembolism = 1 (%)	11 (0.9)	11 (1.0)	0.002
anti-diabetes drugs = 1 (%)	225 (19.3)	203 (17.7)	0.040
antiarrhythmic drugs = 1 (%)	428 (36.7)	463 (40.4)	0.077
beta-blockering agents = 1 (%)	522 (44.8)	529 (46.2)	0.029

Figure A3. Basic characteristics of the subcohort of intracranial hemorrhage before Propensity Score Matching.

After Propensity Score Matching

	NOAC	Warfarin	SMD
n	784	784	
ARBs/ACEIs = 1 (%)	312 (39.8)	334 (42.6)	0.057
Acute myocardial infarction = 1 (%)	11 (1.4)	13 (1.7)	0.021
Age 65-69 = 1 (%)	145 (18.5)	146 (18.6)	0.003
Age 70-74 = 1 (%)	142 (18.1)	134 (17.1)	0.027
Age 75-79 = 1 (%)	134 (17.1)	131 (16.7)	0.010
Age <65 = 1 (%)	159 (20.3)	171 (21.8)	0.038
Age >=80 = 1 (%)	204 (26.0)	202 (25.8)	0.006
Antiplatelet drugs = 1 (%)	343 (43.8)	364 (46.4)	0.054
Coagulation deficiency = 1 (%)	1 (0.1)	1 (0.1)	<0.001
Diabetes = 1 (%)	138 (17.6)	153 (19.5)	0.049
Digoxin = 1 (%)	99 (12.6)	111 (14.2)	0.045
Dihydropyridine calcium channel blockers = 1 (%)	236 (30.1)	257 (32.8)	0.058
GI bleeding = 1 (%)	24 (3.1)	14 (1.8)	0.083
Gender = 1 (%)	327 (41.7)	331 (42.2)	0.010
H2 receptor antagonist = 1 (%)	74 (9.4)	82 (10.5)	0.034
Heart failure = 1 (%)	125 (15.9)	126 (16.1)	0.003
Hypertension = 1 (%)	344 (43.9)	354 (45.2)	0.026
Ischemic stroke = 1 (%)	64 (8.2)	70 (8.9)	0.027
Liver disease = 1 (%)	30 (3.8)	21 (2.7)	0.065
NSAIDs = 1 (%)	134 (17.1)	132 (16.8)	0.007
Non-dihydropyridine calcium channel blockers = 1 (%)	133 (17.0)	132 (16.8)	0.003
Other antacids = 1 (%)	275 (35.1)	283 (36.1)	0.021
Peptic ulcer disease = 1 (%)	37 (4.7)	43 (5.5)	0.035
Peripheral vascular disease = 1 (%)	26 (3.3)	27 (3.4)	0.007
Proton-pump inhibitor = 1 (%)	135 (17.2)	145 (18.5)	0.033
Renal disease = 1 (%)	28 (3.6)	24 (3.1)	0.028
Statins = 1 (%)	150 (19.1)	170 (21.7)	0.063
Transient ischemic attack = 1 (%)	16 (2.0)	15 (1.9)	0.009
Venous Thromboembolism = 1 (%)	10 (1.3)	10 (1.3)	<0.001
anti-diabetes drugs = 1 (%)	131 (16.7)	141 (18.0)	0.034
antiarrhythmic drugs = 1 (%)	280 (35.7)	302 (38.5)	0.058
beta-blockering agents = 1 (%)	343 (43.8)	347 (44.3)	0.010

Figure A4. Basic characteristics of the subcohort of intracranial hemorrhage after Propensity Score Matching.

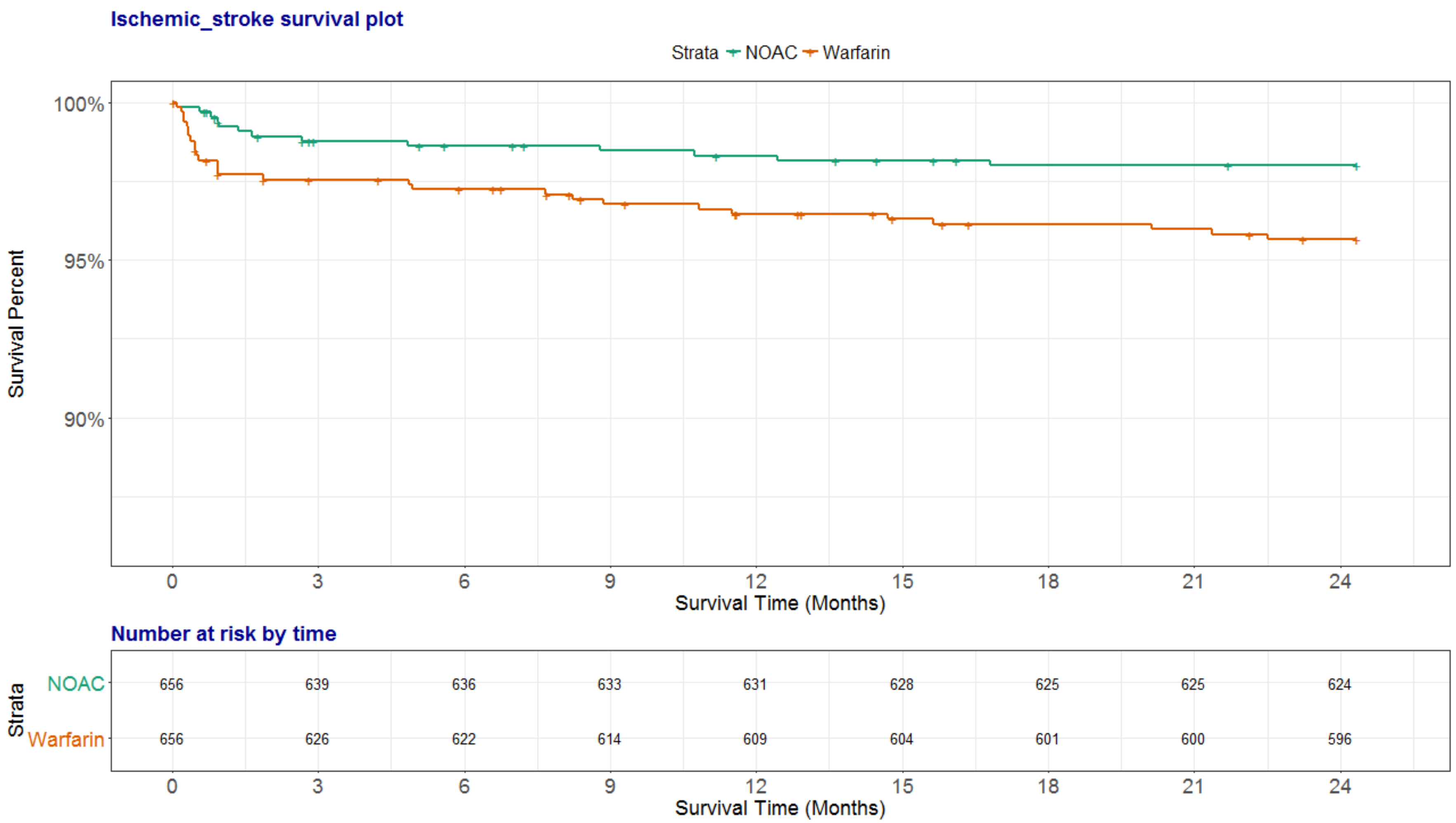

Figure A5. Kaplan–Meier Survival Plots of ischemic stroke with ITT analysis

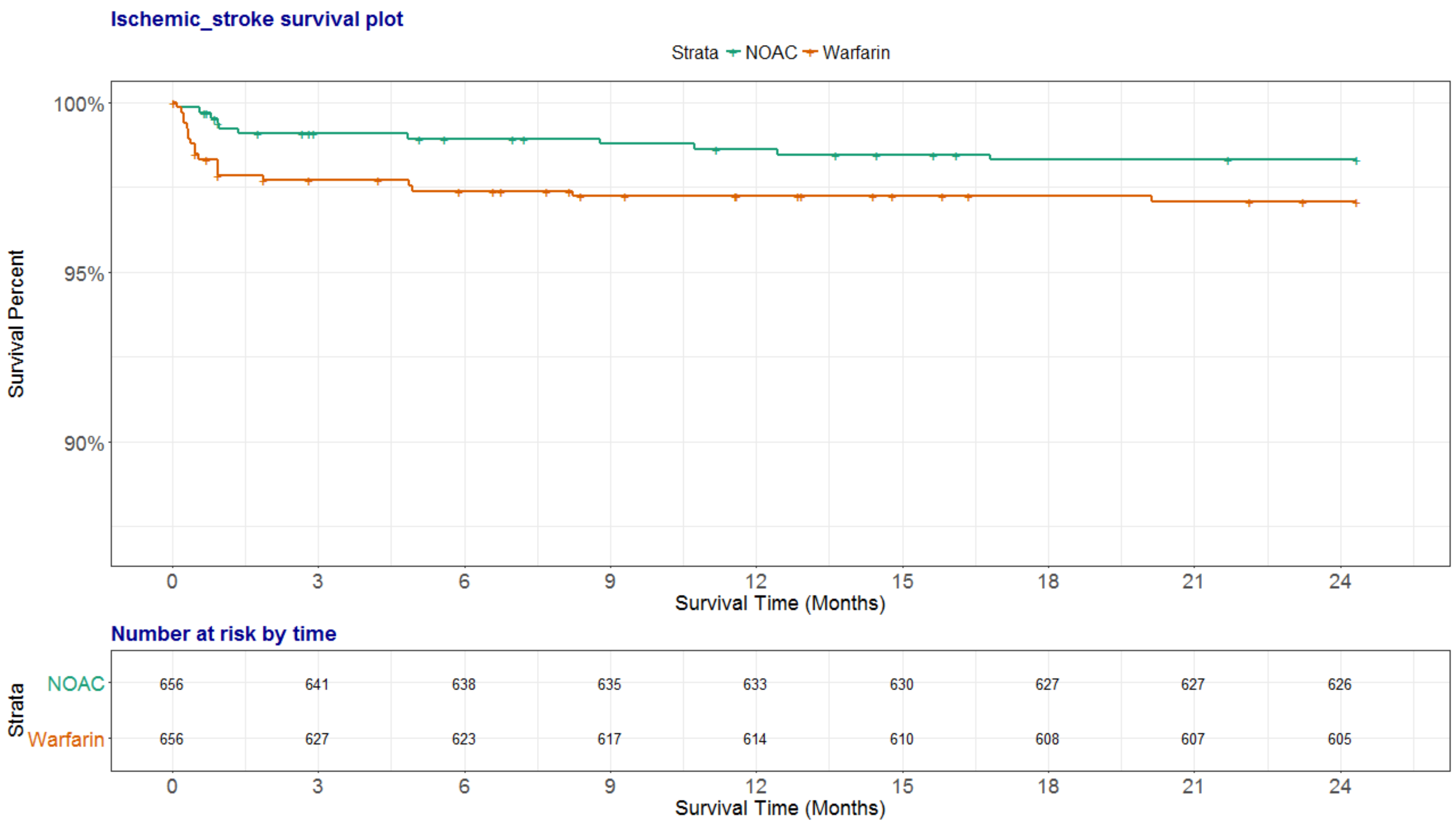

Figure A6. Kaplan–Meier Survival Plots of ischemic stroke with AT analysis

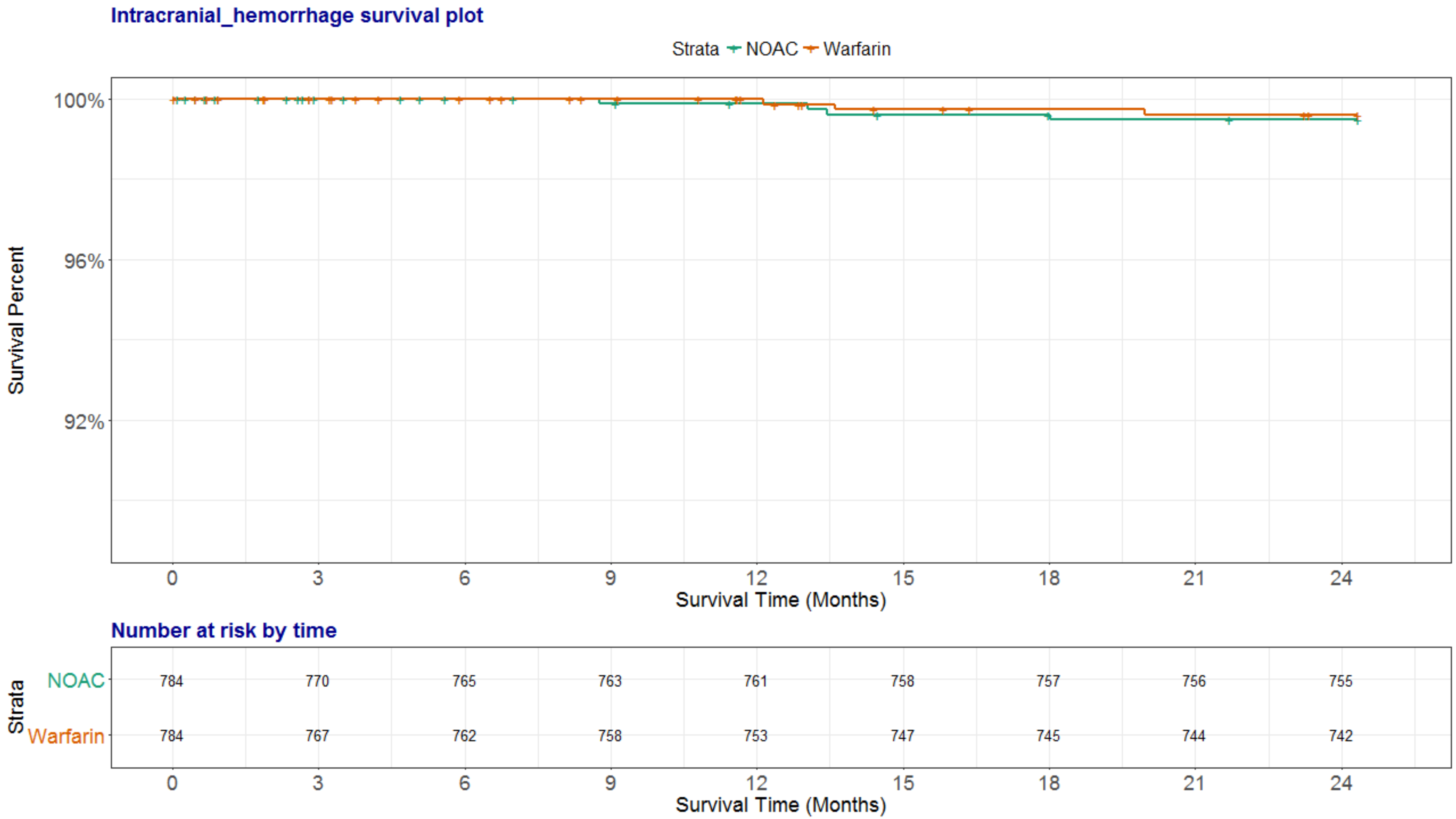

Figure A7. Kaplan–Meier Survival Plots of Intracranial hemorrhage with ITT analysis

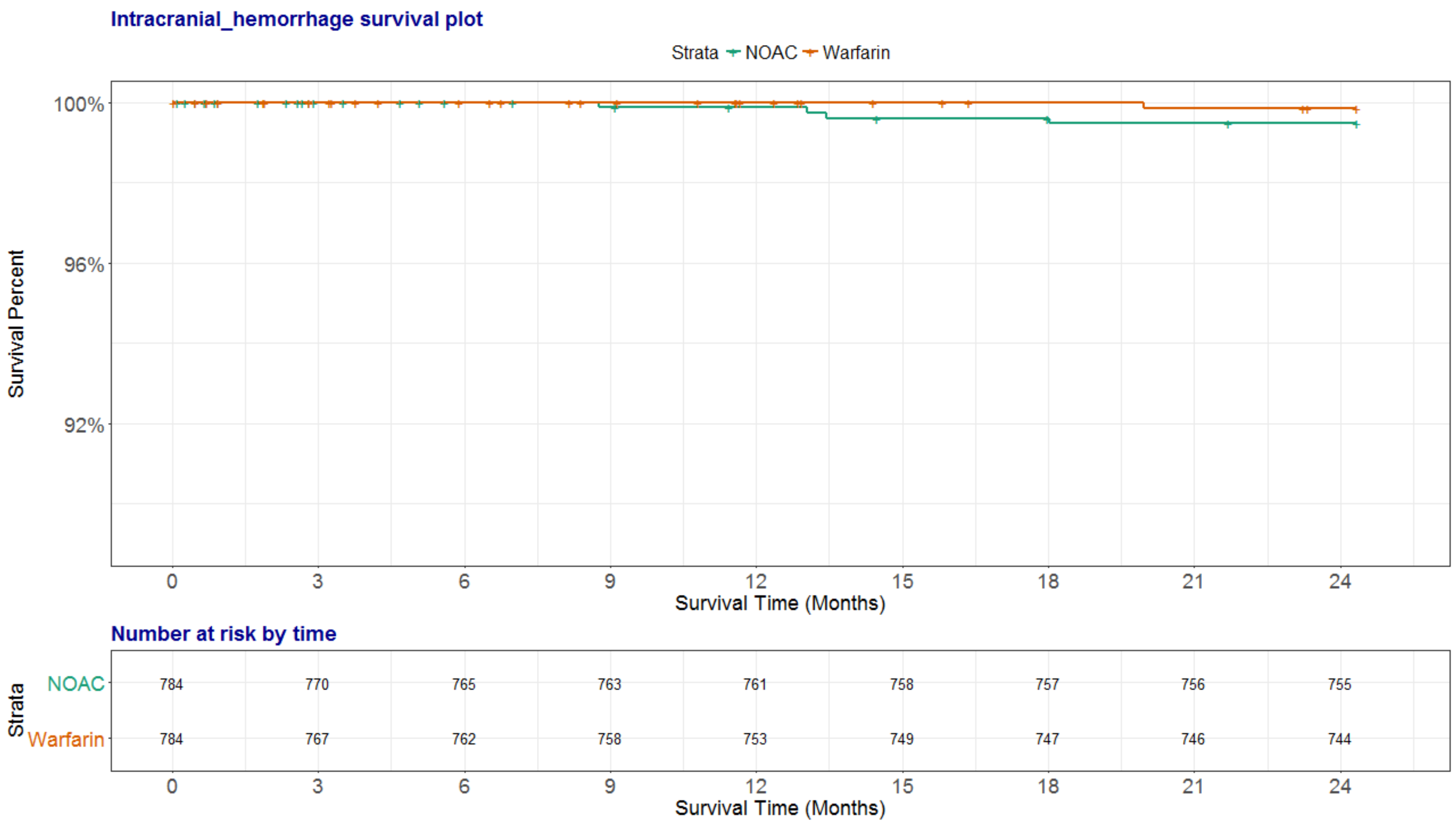

Figure A8. Kaplan–Meier Survival Plots of Intracranial hemorrhage with AT analysis
